# Global prevalence of restless legs syndrome among hemodialysis patients: A systematic review and meta‐analysis

**DOI:** 10.1002/brb3.3378

**Published:** 2024-01-11

**Authors:** Xu‐Hua Zhou, Yuan Liu, Xin‐Rui Zhang, Cong Wang, Shan‐Shan Liu, Yan Jiang

**Affiliations:** ^1^ Evidence‐Based Nursing Center, West China Hospital Sichuan University/West China School of Nursing, Sichuan University Chengdu Sichuan P. R. China; ^2^ Nursing Department, West China Hospital Sichuan University/West China School of Nursing Sichuan University Chengdu Sichuan P. R. China

**Keywords:** hemodialysis, meta‐analysis, prevalence, restless legs syndrome, worldwide

## Abstract

**Objectives**: Restless legs syndrome (RLS) is a common complaint in patients undergoing hemodialysis (HD). Despite the fact that the estimated prevalence of RLS among HD patients is widely reported, these results varied significantly in the relevant literature. Due to this limitation, the aim of this study was to determine the global prevalence of RLS among HD patients.

**Methods**: This systematic review was conducted and reported in accordance with the Preferred Reporting Items for Systematic Reviews and Meta‐analyses 2020 statement. We searched the electronic databases: Cochrane Library, PubMed, Embase, Web of Science, Scopus, Cumulative Index to Nursing and Allied Health Literature, China Knowledge Resource Integrated Database, Wanfang Database, Chinese Biomedical Database, and Weipu Database. A random effects model was employed to calculate pooled prevalence rates.

**Results**: The global pooled prevalence of RLS in HD patients was 27.2% (95% CI: 24.8–29.7). Stratified analyses demonstrated that included studies with sample size <100 had the highest pooled prevalence of RLS. The prevalence of RLS using clinical interviews and questionnaires was 28.7% (95% CI: 25.2–32.2) and 25.9% (95% CI: 22.8–29.1), respectively. RLS prevalence is higher in females (29.7%, 95% CI: 26.2–33.2) HD patients than in males (23.5%, 95% CI: 20.9–26.0), and the African region has the highest prevalence in the world when the diagnostic criteria were restricted to the 2003 version of International RLS Study Group criteria, the prevalence of RLS was highest (28.9%, 95% CI: 25.9–31.9).

**Conclusion**: Our results revealed a high RLS prevalence in HD patients worldwide. However, the prevalence of RLS among HD patients varied significantly based on sample size, data collection method, gender, diagnostic criteria, and geographical region.

## 1 INTRODUCTION

Restless legs syndrome (RLS), a sleep‐related sensory‐motor disorder, is characterized by intolerable discomforts such as insect crawling, pins and needles, and itching in the deep part of the calf between the knee and ankle joints (Castillo‐Torres et al., 2018; de Menezes et al., 2018; Wang et al., 2021). RLS has a distinctive circadian rhythm and is relieved or eliminated by physical activity (Lin et al., 2019; Yaseen et al., 2022). It can be primary, hereditary, or secondary to a variety of chronic conditions, such as end‐stage renal disease (ESRD), Parkinson's disease, and iron deficiency anemia (Ghanei Gheshlagh et al., 2017; Sagheer et al., 2018; Yang et al., 2018). Of particular note, patients on hemodialysis (HD) for ESRD are amongst the groups most at risk of secondary RLS (Hamed et al., 2023; Örsal et al., 2017; Turgay et al., 2018).

ESRD is a clinical syndrome involving an irreversible deterioration in kidney function, characterized by the kidney's inability to efficiently filter waste and maintain electrolyte balance (Bhandari et al., 2022; Cockwell et al., 2020). HD is the most common renal replacement therapy for ESRD patients and is effective in relieving their clinical symptoms (Johnson & Meyer, 2018). However, patients on HD are more vulnerable to RLS due to ferritin loss and changes in phosphate and parathyroid hormone levels, suggesting that HD is a significant risk factor for RLS (Huzmeli et al., 2018; Ramachandran et al., 2018). Moreover, HD ‐related RLS is associated with a variety of physical and psychological disorders, such as insomnia, fatigue, anxiety, and depression, which seriously affect patients’ prognosis and quality of life and even lead to an increased risk of cardiovascular events and death (Mousavi et al., 2021; Stergiannis et al., 2020; Tuncel et al., 2011, 2019; Tsai et al., 2019; Yang et al., 2019; Zhang et al., 2020).

Comprehending the current epidemiology of RLS in HD patients is crucial for clinical researchers to devise appropriate prevention and treatment strategies. RLS secondary to HD has attracted a great deal of attention from researchers around the world in recent decades (Brzuszek et al., 2022; Chavoshi et al., 2015; Hasheminasab Zaware et al., 2016). Based on the International RLS Study Group (IRLSSG) diagnostic criteria, various studies from different countries have reported a prevalence of RLS in HD patients ranging from 5% to 70% (Bathla et al., 2016; Hui et al., 2002; Pizza et al., 2012; Xiao et al., 2017). A published systematic review has reported on the prevalence of RLS among HD patients. After careful consideration, we have identified some common limitations as follows: (1) Few studies included in the analysis due to language and electronic database limitations; (2) failure to rationally divide subgroups to explore differences in the prevalence of RLS among HD patients with different characteristics. These substantial limitations highlight the need for a comprehensive meta‐analysis to determine more reliable estimates of the prevalence of RLS in the HD population and to inform the development of screening and intervention strategies. Therefore, the present study systematically reviewed the publications on the prevalence of RLS among HD patients to answer the following questions:

1. What is the global prevalence of RLS among HD patients?
2. Is the prevalence of RLS affected by sample size, gender, data collection methods, diagnostic criteria, or geographical region?
3. Is the prevalence of RLS moderated by sample size, gender, data collection methods and diagnostic criteria, and geographical region?

## 2 METHODS

After adhering to the Preferred Reporting Items for Systematic Reviews and Meta‐analyses criteria (Moher et al., 2015) Supplementary section (S1), we conducted this systematic review and meta‐analysis. The review protocol has been duly registered in the International Prospective Register of Systematic Reviews (CRD42020194427).

### 2.1 Search strategy

A comprehensive concept‐based literature search was conducted in 10 electronic academic databases (Cochrane Library; PubMed; Embase; Web of Science; Scopus; Cumulative Index to Nursing and Allied Health Literature; China Knowledge Resource Integrated Database; Wanfang Database; Chinese Biomedical Database; and Weipu Database) from the inception of the respective database to August 10, 2023, with no language restrictions. The initial search terms included “dialysis,” “hemodialysis,” “restless legs syndrome,” “Willis‐Ekbom Disease,” and “Wittmaack‐Ekbom syndrome.” In each database, keywords and medical subject headings were combined by using Boolean operators such as “and” and “or.” The reference lists of included studies and review articles were hand‐searched to identify any additional pertinent studies. The detailed search strategy is attached in presented in the Supplementary section (S1).

### 2.2 Study selection

Two reviewers (XHZ and YL) carried out the selection of studies for inclusion in the review, which consisted of title/abstract screening with subsequent full‐text checking. Any disagreements were resolved by consultation with a senior reviewer (CW). To be eligible for inclusion, articles had to meet the following criteria: (1) studies with cross‐sectional or longitudinal design; (2) participants were patients with ESRD receiving HD; (3) studies reporting the prevalence of RLS in HD patients; (4) researches that use clinical interview or questionnaire to collect data and diagnose RLS using criteria developed by the IRLSSG. The following kinds of studies were excluded: (1) reviews, case reports, comments, editorials, or conference abstracts; (2) case‐control studies; and (3) articles with no access to full text or duplicate data. If the same total population was used in multiple publications, only the dataset with the largest sample was selected.

### 2.3 Data extraction

To ensure the rigor of the process of extracting data from the included studies, a predesigned electronic Excel spreadsheet was created. Data were extracted from all eligible studies independently by two reviewers (XHZ and YL), who then cross‐checked the data for accuracy after the extraction was complete. The following data were extracted from each study: first author; year of publication; country of origin; study design; sample size; proportion of females in the total sample; data collection method; diagnostic criteria; and the prevalence of RLS among HD patients. Some authors were contacted to fill in missing information where possible.

### 2.4 Quality assessment

The risk of bias in the included studies was assessed and cross‐checked independently by two reviewers. In case of disagreement, a third reviewer was consulted to make the final decision. Cross‐sectional studies were scored using the Agency for Healthcare Research and Quality (AHRQ), which has 11 items, with a score of 1 if each item was answered “yes,” and 0 if “no” or “unclear” (Chou et al., 2018). The total score for the AHRQ ranges from 8 to 11 points for high quality, 4 to 7 points for medium quality, and 0 to 3 points for low quality. The Newcastle‐Ottawa Scale (NOS) was employed to appraise the methodological quality of the included cohort studies (Xu et al., 2022). According to the scoring criteria of this table, the overall rating is based on the cut‐off values of 1–3, 4–6, and 7–9, representing high, moderate, and low risk of bias, respectively.

### 2.5 Statistical analysis

Statistical analyses were conducted by Stata 14.2 software and a *p* value <.05 (two‐sided test) was considered to indicate statistical significance. The main statistical indicators were the prevalence of RLS and the corresponding 95% confidence intervals (CIs). In our meta‐analysis, heterogeneity among included studies was assessed using the *I* ^2^ statistic, which was classified into three categories based on *I* ^2^ values: 25%–50% (low), 50%–75% (moderate), and ≥75% (high) (Higgins et al., 2003). A random‐effects model was applied to calculate the pooled prevalence of RLS if significant heterogeneity was detected; otherwise, a fixed‐effects model was employed. Subgroup analysis and meta‐regression were utilized to identify potential moderators between study heterogeneity. We conducted subgroup analysis and univariate and multivariate meta‐regression to explore the variability of RLS prevalence among HD patients due to sample size, data collection method, gender, diagnostic criteria, and geographical region.

In the present study, publication bias was evaluated by using a visualized funnel plot and objectively using Egger's linear regression test. Additionally, to address asymmetry in the funnel plot potentially resulting from publication bias, we conducted a trim‐and‐fill analysis. The robustness of the results was examined through a sensitivity analysis approach that removed any single study.

## 3 RESULTS

### 3.1 Search results summary

**FIGURE 1:**
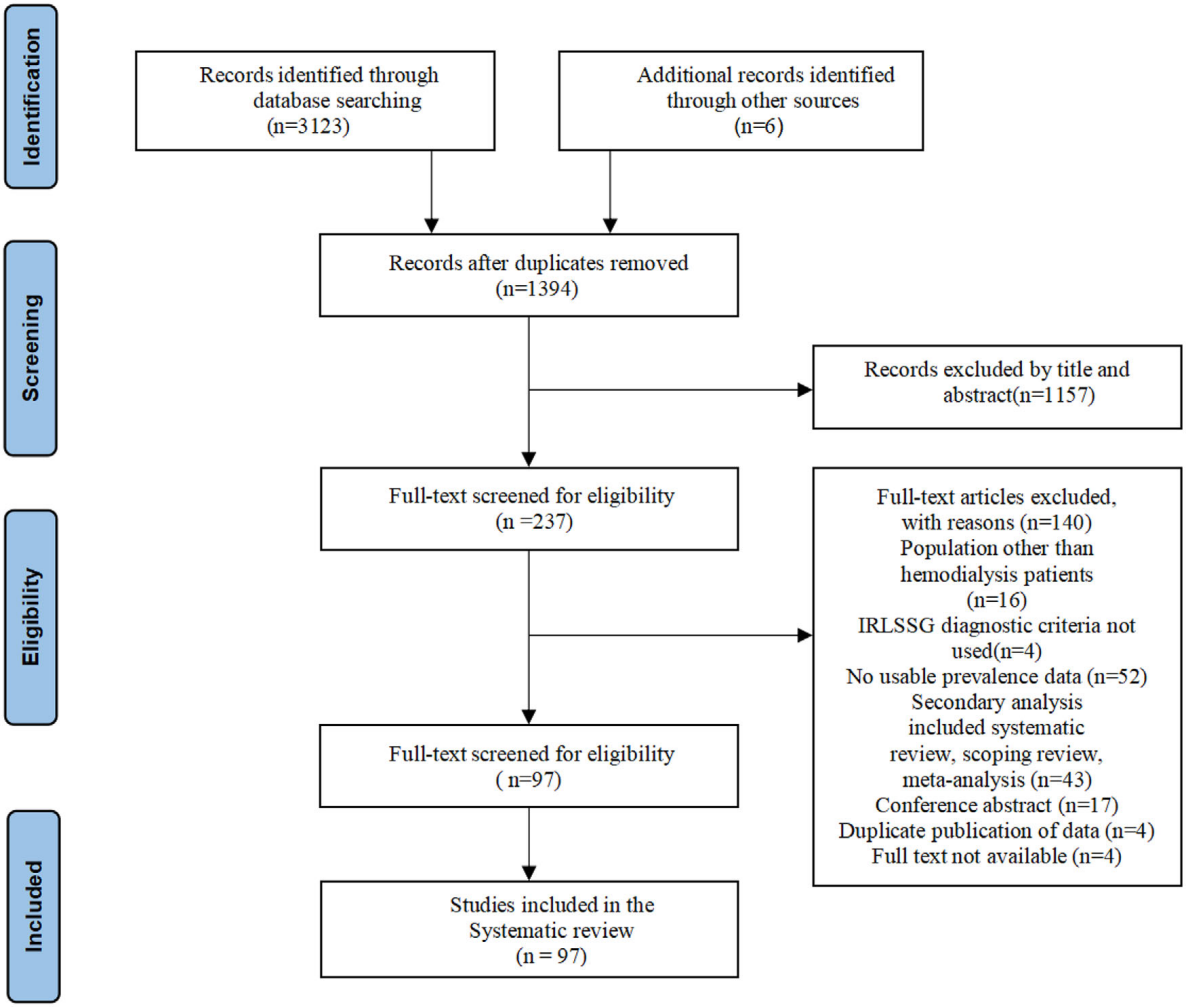
Flow diagram of study selection.

The initial electronic literature search resulted in 3123 articles, and an additional 6 studies were yielded from reference lists. After removing 1735 duplicates, a total of 1394 articles underwent screening based on their titles and abstracts, resulting in 237 studies that met the validation criteria. Of the 237 articles, a cluster of 97 studies were finally included in this study after a full‐text review. The flowchart of the selection process is shown in Figure 1.

### 3.2 Characteristics of included studies

Detailed characteristics of the 97 studies included in this review are summarized in Table 1. The 97 articles included in this study were published between 1998 and 2023, and they encompass studies conducted in 21 different countries. The distribution of these studies is as follows: China (35), Iran (10), Turkey (10), Italy (6), Brazil (5), India (4), Pakistan (4), Egypt (3), Japan (3), Hungary (2), Greece (2), UK (2), Serbia (2), Saudi Arabia (2), Australia (1), Croatia (1), Germany (1), Korea (1), Morocco (1), Netherlands (1), and Syria (1). Ninety‐five studies were cross‐sectional surveys, and two were cohort studies. The pooled number of patients included in the study was 23,248, with sample sizes ranging from 30 to 3025 in a single study. Forty‐five studies used clinical interviews to assess RLS symptoms among HD patients, and the remaining 52 used questionnaires. Of the literature utilizing the IRLSSG diagnostic criteria, 19 studies used the 1995 version, 61 studies used the 2003 version, 4 studies used the 2012 version, and 13 studies used the 2014 version.

**TABLE 1:** Characteristics of the included studies.

References	Country	Study design	Sample size	Female (%)	Method for data collection	Diagnostic criteria	Prevalence of RLS (%)	Quality assessment
Al‐Jahdali et al. (2009)	Saudi Arabia	Cross‐sectional	188	Unclear	Interview	IRLSSG (1995)	46.3	6
Araujo et al. (2010)	Brazil	Cross‐sectional	400	41.00	Interview	IRLSSG (2003)	21.5	5
Bastos et al. (2007)	Brazil	Cross‐sectional	100	41.00	Interview	IRLSSG (2003)	48.0	5
Bathla et al. (2016)	India	Cross‐sectional	194	41.80	Interview	IRLSSG (2014)	5.2	5
Beladi‐Mousavi et al. (2015)	Iran	Cross‐sectional	139	43.20	Interview	IRLSSG (2003)	15.8	7
Bhagawati et al. (2019)	India	Cross‐sectional	185	25.70	Questionnaire	IRLSSG (2003)	29.2	6
Brzuszek et al. (2022)	UK	Cross‐sectional	106	41.50	Interview	IRLSSG (2003)	36.8	6
Chavoshi et al. (2015)	Iran	Cross‐sectional	397	34.00	Interview	IRLSSG (2003)	31.7	6
Chen et al. (2018)	China	Cross‐sectional	115	55.70	Questionnaire	IRLSSG (2012)	20.9	6
Chu et al. (2014)	Australia	Cross‐sectional	85	63.50	Questionnaire	IRLSSG (1995)	24.7	4
Cirignotta et al. (2002)	Italy	Cross‐sectional	114	Unclear	Questionnaire	IRLSSG (1995)	33.3	5
Collado‐Seidel et al. (1998)	Germany	Cross‐sectional	136	38.20	Interview	IRLSSG (1995)	23.5	4
de Menezes et al. (2018)	Brazil	Cross‐sectional	241	41.50	Interview	IRLSSG (2003)	17.4	6
Dikici et al. (2014)	Turkey	Cross‐sectional	246	49.80	Interview	IRLSSG (1995)	45.9	6
Du et al. (2017)	China	Cross‐sectional	307	43.30	Questionnaire	IRLSSG (2012)	12.1	7
Giannaki et al. (2011)	Greece	Cross‐sectional	70	27.10	Interview	IRLSSG (2003)	42.9	6
Gigli et al. (2004)	Italy	Cross‐sectional	362	Unclear	Questionnaire	IRLSSG (2003)	32.0	6
Goffredo et al. (2003)	Brazil	Cross‐sectional	176	39.20	Interview	IRLSSG (1995)	14.8	6
Guan et al. (2016)	China	Cross‐sectional	186	52.70	Questionnaire	IRLSSG (2014)	16.1	6
Hamed et al. (2021)	Egypt	Cross‐sectional	400	34.50	Interview	IRLSSG (2014)	26.0	7
Higuchi et al. (2015)	Japan	Cross‐sectional	157	29.30	Interview	IRLSSG (2003)	22.3	6
Huang et al. (2017)	China	Cross‐sectional	119	43.70	Questionnaire	IRLSSG (2014)	17.6	5
Hui et al. (2002)	China	Cross‐sectional	43	37.20	Questionnaire	IRLSSG (1995)	69.8	5
Huzmeli et al. (2018)	Turkey	Cross‐sectional	75	53.30	Questionnaire	IRLSSG (2003)	44.0	6
Ibrahim et al. (2011)	Egypt	Cross‐sectional	264	44.30	Questionnaire	IRLSSG (2003)	56.4	6
Kawauchi et al. (2006)	Japan	Cross‐sectional	228	39.80	Questionnaire	IRLSSG (1995)	23.2	6
Kaya et al. (2015)	Turkey	Cross‐sectional	232	43.50	Interview	IRLSSG (2014)	11.6	5
Kim et al. (2008)	Korea	Cross‐sectional	164	43.90	Questionnaire	IRLSSG (1995)	28.0	6
Kutlu et al. (2018)	Turkey	Cross‐sectional	237	46.80	Interview	IRLSSG (1995)	18.6	5
La Manna et al. (2011)	Italy	Cohort	100	37.00	Interview	IRLSSG (2003)	31.0	7
Li et al. (2023)	China	Cross‐sectional	169	42.00	Questionnaire	IRLSSG (2014)	39.1	6
Lin et al. (2013)	China	Cross‐sectional	1130	43.20	Interview	IRLSSG (2003)	25.3	7
Lin et al. (2018)	China	Cross‐sectional	304	37.50	Questionnaire	IRLSSG (2012)	14.1	6
Lin et al. (2019)	China	Cross‐sectional	137	46.00	Questionnaire	IRLSSG (2003)	20.4	8
Liu et al. (2006)	China	Cross‐sectional	108	66.70	Questionnaire	IRLSSG (2003)	13.0	5
Ma et al. (2021)	China	Cross‐sectional	186	43.50	Questionnaire	IRLSSG (2003)	39.3	5
Meng et al. (2017)	China	Cross‐sectional	301	41.90	Questionnaire	IRLSSG (2003)	25.2	6
Meng et al. (2021)	China	Cross‐sectional	382	48.20	Interview	IRLSSG (2003)	33.5	4
Merlino et al. (2006)	Italy	Cross‐sectional	883	38.80	Questionnaire	IRLSSG (1995)	18.4	6
Merlino et al. (2012)	Italy	Cross‐sectional	58	Unclear	Interview	IRLSSG (2003)	19.0	5
Mucsi et al. (2004)	Hungary	Cross‐sectional	78	Unclear	Questionnaire	IRLSSG (2003)	15.4	5
Mucsi et al. (2005)	Hungary	Cross‐sectional	333	42.00	Questionnaire	IRLSSG (2003)	13.5	6
Naini et al. (2012)	Iran	Cross‐sectional	45	44.40	Questionnaire	IRLSSG (2003)	35.5	6
Neves et al. (2017)	Brazil	Cross‐sectional	101	46.50	Interview	IRLSSG (2014)	28.7	6
Nikić et al. (2007)	Serbia	Cross‐sectional	173	37.00	Questionnaire	IRLSSG (1995)	17.9	5
Örsal et al. (2017)	Turkey	Cross‐sectional	244	49.20	Interview	IRLSSG (2003)	15.6	4
Pan et al. (2006)	china	Cross‐sectional	171	34.90	Questionnaire	IRLSSG (2003)	25.2	5
Pavan et al. (2014)	India	Cross‐sectional	50	26.00	Interview	IRLSSG (2003)	28.0	5
Pizza et al. (2012)	Italy	Cross‐sectional	162	35.20	Interview	IRLSSG (2003)	31.5	6
Rafie et al. (2016)	Iran	Cross‐sectional	137	46.70	Interview	IRLSSG (2003)	36.5	6
Ramachandran et al. (2018)	India	Cross‐sectional	116	31.90	Questionnaire	IRLSSG (2014)	10.3	6
Razeghi et al. (2012)	Iran	Cross‐sectional	108	42.60	Questionnaire	IRLSSG (2003)	32.4	5
Rijsman et al. (2004)	Netherlands	Cross‐sectional	30	Unclear	Interview	IRLSSG (2003)	46.7	5
Rohani et al. (2014)	Iran	Cross‐sectional	163	36.80	Interview	IRLSSG (2003)	37.4	6
Sabry et al. (2010)	Egypt	Cross‐sectional	88	Unclear	Questionnaire	IRLSSG (1995)	42.0	6
Salman et al. (2011)	Syria	Cross‐sectional	123	42.40	Interview	IRLSSG (1995)	20.3	5
Samavat et al. (2017)	Iran	Cross‐sectional	235	43.40	Interview	IRLSSG (2003)	23.4	6
Zadeh Saraji et al. (2017)	Iran	Cross‐sectional	260	39.60	Interview	IRLSSG (2003)	55.0	5
Shaikh et al. (2014)	Pakistan	Cross‐sectional	100	45.00	Interview	IRLSSG (2003)	32.0	5
Shao et al. (2015)	China	Cross‐sectional	113	40.70	Questionnaire	IRLSSG (2003)	19.5	5
Shen et al. (2013)	China	Cross‐sectional	194	31.40	Questionnaire	IRLSSG (2003)	16.0	5
Shen et al. (2018)	China	Cross‐sectional	74	37.80	Questionnaire	IRLSSG (2003)	19.6	6
Shi et al. (2015)	China	Cross‐sectional	186	40.30	Questionnaire	IRLSSG (2003)	21.5	5
Shi et al. (2018)	China	Cross‐sectional	220	53.60	Questionnaire	IRLSSG (2014)	48.2	6
Siddiqui et al. (2005)	UK	Cross‐sectional	277	48.00	Interview	IRLSSG (1995)	45.8	6
Sladojević et al. (2012)	Serbia	Cross‐sectional	96	38.50	Questionnaire	IRLSSG (2003)	39.6	4
Soumeila et al. (2015)	Morocco	Cross‐sectional	84	61.90	Interview	IRLSSG (2003)	41.7	5
Soyoral et al. (2010)	Turkey	Cross‐sectional	76	44.70	Interview	IRLSSG (1995)	14.5	4
Stefanidis et al. (2013)	Greece	Cross‐sectional	579	40.70	Interview	IRLSSG (2003)	26.6	8
Sultan et al. (2022)	Pakistan	Cross‐sectional	112	37.50	Interview	IRLSSG (2003)	38.4	6
Takaki et al. (2003)	Japan	Cross‐sectional	490	28.80	Questionnaire	IRLSSG (1995)	12.2	6
Tang et al. (2014)	China	Cross‐sectional	424	40.30	Questionnaire	IRLSSG (2003)	36.6	7
Tekdöş et al. (2015)	Turkey	Cross‐sectional	118	53.10	Interview	IRLSSG (2003)	41.5	5
Telarović et al. (2007)	Croatia	Cross‐sectional	82	35.40	Interview	IRLSSG (2003)	59.8	6
Tufekci et al. (2021)	Turkey	Cross‐sectional	72	55.60	Questionnaire	IRLSSG (2003)	50.0	7
Tuncel et al. (2011)	Turkey	Cross‐sectional	81	49.40	Interview	IRLSSG (2003)	12.3	4
Tuo et al. (2017)	China	Cross‐sectional	94	40.40	Questionnaire	IRLSSG (2003)	48.9	6
Turgay et al. (2018)	Turkey	Cross‐sectional	360	Unclear	Interview	IRLSSG (2003)	16.9	5
Ul Abideen et al. (2018)	Pakistan	Cross‐sectional	279	48.00	Interview	IRLSSG (2012)	24.0	5
Wali et al. (2015)	Saudi Arabia	Cross‐sectional	355	39.00	Interview	IRLSSG (2003)	19.4	6
Wang et al. (2020)	China	Cross‐sectional	135	50.40	Interview	IRLSSG (2003)	18.5	5
Wang et al. (2023)	China	Cross‐sectional	3025	39.90	Questionnaire	IRLSSG (2014)	8.8	8
Xiao et al. (2013)	China	Cross‐sectional	375	47.50	Questionnaire	IRLSSG (2003)	13.3	5
Xiao et al. (2017)	China	Cross‐sectional	269	30.10	Interview	IRLSSG (2003)	14.5	6
Xu et al. (2015)	China	Cross‐sectional	137	37.20	Questionnaire	IRLSSG (2003)	14.6	6
Xu et al. (2023)	China	Cross‐sectional	286	54.90	Questionnaire	IRLSSG (1995)	13.3	6
Yang et al. (2019)	China	Cohort	578	37.20	Questionnaire	IRLSSG (2003)	14.4	8
Yaseen et al. (2022)	Pakistan	Cross‐sectional	150	58.00	Questionnaire	IRLSSG (2014)	26.7	5
Yazdi et al. (2015)	Iran	Cross‐sectional	112	35.70	Questionnaire	IRLSSG (2003)	42.9	4
Hasheminasab Zaware et al. (2016)	Iran	Cross‐sectional	44	52.30	Interview	IRLSSG (2003)	54.5	5
Zeng et al. (2022)	China	Cross‐sectional	247	27.90	Questionnaire	IRLSSG (2014)	19.8	6
Zhang et al. (2007)	China	Cross‐sectional	67	34.30	Questionnaire	IRLSSG (2003)	13.4	5
Zhang et al. (2016)	China	Cross‐sectional	115	42.60	Questionnaire	IRLSSG (2003)	33.9	6
Zhang et al. (2020)	China	Cross‐sectional	354	44.10	Questionnaire	IRLSSG (2014)	40.7	7
Zhang et al. (2022)	China	Cross‐sectional	194	43.80	Questionnaire	IRLSSG (2003)	29.4	6
Zhang et al. (2022)	China	Cross‐sectional	527	45.40	Questionnaire	IRLSSG (2003)	7.2	5
Zhong et al. (2012)	China	Cross‐sectional	126	42.90	Questionnaire	IRLSSG (1995)	16.7	5

Abbreviations: IRLSSG, International RLS Study Group; RLS, restless legs syndrome.

### 3.3 Quality assessment of included studies

The AHRQ and NOS were employed to conduct the methodological evaluation of the included cross‐sectional and cohort studies, respectively. Quality assessment scores for cross‐sectional studies ranged from 3 to 8; out of the 95 cross‐sectional studies included, three were categorized as “high quality,” whereas the remaining 92 were categorized as “moderate quality” (Table S1). Both cohort studies were identified as having a low risk of bias (Table S2).

### 3.4 Prevalence of RLS among hemodialysis patients

**FIGURE 2:**
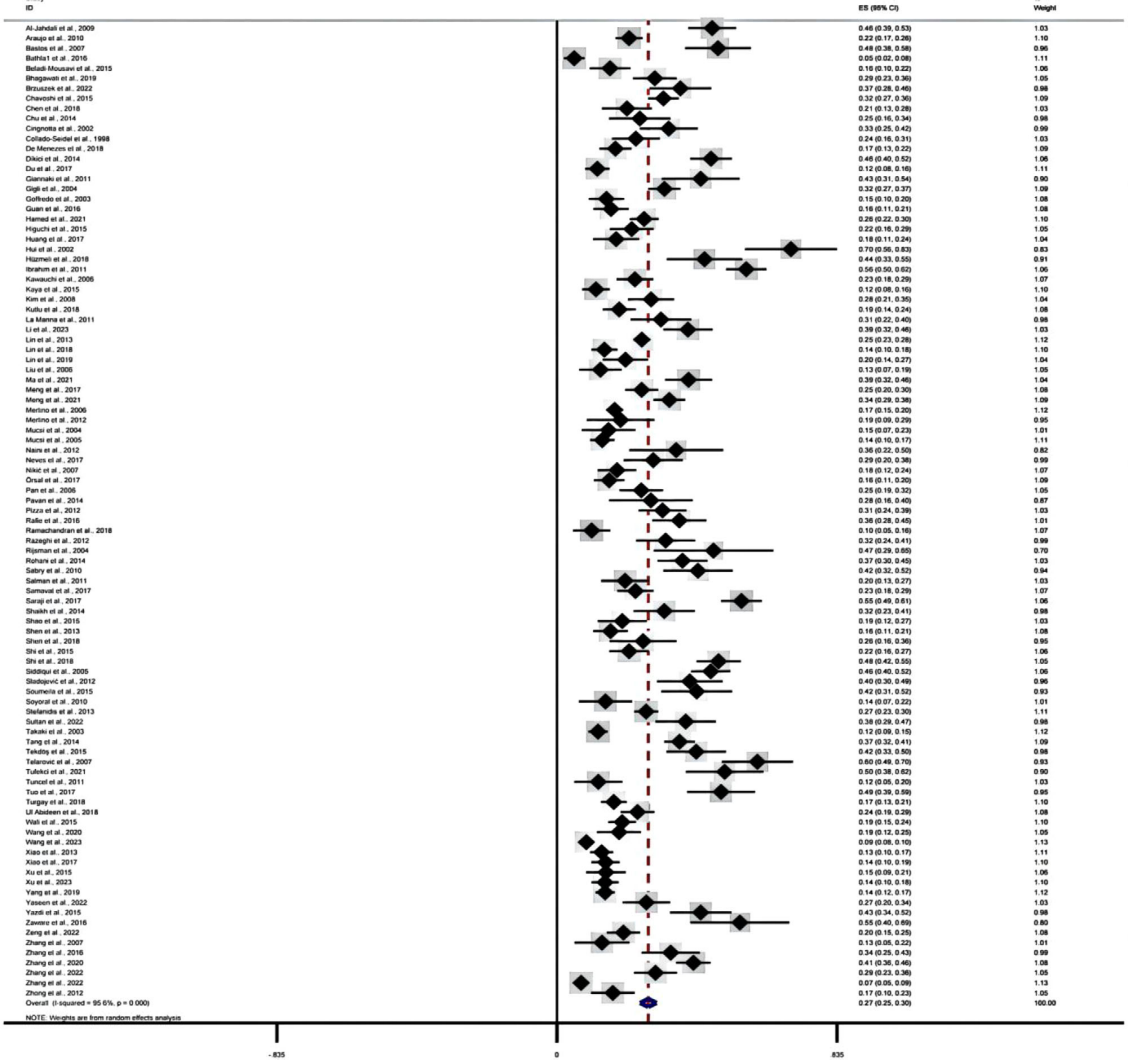
Forest plot overall restless legs syndrome (RLS) prevalence among hemodialysis patients.

All included studies (*n* = 97) investigated and reported the prevalence of RLS inHD patients, and the prevalence ranged from 5.2% to 69.8%. Due to the substantial heterogeneity detected, the meta‐analysis was performed using a random‐effects model. The random‐effects model indicated that the pooled prevalence of RLS among HD patients was 27.2% (95% CI: 24.8–29.7, *I* ^2^ = 95.6%, *p* < .001) (Figure 2).

### 3.5 Subgroup analysis and meta‐regression analysis

Considerable heterogeneity was detected across the included studies when pooling RLS prevalence among HD patients. Thus, a subgroup analysis was undertaken stratifying by sample size, data collection method, gender, diagnostic criteria, and geographical region to investigate the sources of heterogeneity. Subgroup analysis of RLS was carried out and summarized in Table 2. When assessed by sample size, the estimated pooled prevalence of RLS for sample sizes <100, 100–300, and >300 was 35.9%, 27.0%, and 21.2%, respectively (Figure S1). In the subgroup analysis based on the data collection method, the pooled prevalence of RLS was higher in the clinical interview group (28.7%) than in the questionnaire group (25.9%) (Figure S2). The prevalence of RLS among patients on HD was 23.5% in males and 29.7% in females, respectively (Figure S3). When analyzed using different diagnostic criteria, the prevalence of RLS varied: IRLSSG (1995), (27.1%); IRLSSG (2003), (28.9%); IRLSSG (2012), (17.4%); IRLSSG (2014), (22.7%) (Figure S4). After analyzing subgroups by geographic region, we clarified the prevalence of RLS among HD patients in various regions of the world: Asia (25.7%), Africa (39.0%), Europe (29.8%), Oceania (24.7%), and South America (30.3%) (Figure S5).

**TABLE 2:** Subgroup analyses by the sample size, data collection method, gender, diagnostic criteria, and geographical region.

Subgroups	Number of included studies	Prevalence of RLS (%)	95% CI	*I* ^2^ (%)	*p* Value
Sample size					
<100	20	35.9	28.4–43.4	90.4	<.001
100–300	56	27.0	23.7–30.4	93.9	<.001
>300	21	21.2	17.2–25.1	97.2	<.001
Data collection method					
Clinical interview	45	28.7	25.2–32.2	93.9	<.001
Questionnaire	52	25.9	22.8–29.1	95.8	<.001
Gender					
Male	66	23.5	20.9–26.0	90.9	<.001
Female	66	29.7	26.2–33.2	92.8	<.001
Diagnostic criteria					
IRLSSG (1995)	19	27.1	21.8–32.5	94.4	<.001
IRLSSG (2003)	61	28.9	25.9–31.9	94.1	<.001
IRLSSG (2012)	4	17.4	11.8–23.0	82.2	.001
IRLSSG (2014)	13	22.7	16.0–29.5	97.2	<.001
Geographical region					
Asia	70	25.7	23.0–28.5	95.7	<.001
Africa	4	39.0	17.4–60.6	97.0	<.001
Europe	17	29.8	24.1–35.5	94.0	<.001
Oceania	1	24.7	15.5–33.9		
South America	5	30.3	23.2–37.3	86.5	<.001

Abbreviations: CI, confidence interval; IRLSSG, International RLS Study Group; RLS, restless legs syndrome.

Meta‐regression analysis was conducted to examine the association between the prevalence of RLS in HD patients and study characteristics, including sample size, method of data collection, gender, diagnostic criteria, and geographical region (Table S3). In the multivariate meta‐regression model, data collection methods, gender, and diagnostic criteria were not statistically significant in explaining the variance in RLS prevalence estimates across studies. However, sample size (*β* = −.07, 95% CI = −0.11 to 0.02, *p* = .002) and geographical region (*β* = −.01, 95% CI = −0.04 to 0.02, *p* = .014) were significant potential moderators of the overall heterogeneity, yielding a model that was capable of explaining 8.94% of the variation between including studies.

### 3.6 Publication bias and sensitivity analysis

Evidence of publication bias in the included studies was revealed through a funnel plot analysis (Figure S6). Furthermore, the results of Egger's test (*t* = 3.69, *p* < .001) provided additional confirmation of publication bias regarding the prevalence of RLS in HD patients (Figure S7). Therefore, we conducted a trim and fill analysis to adjust for this bias. During the analysis, studies included in RLS estimation among HD patients were adjusted. The corrected pooled prevalence estimate of RLS is similar to the unadjusted result, indicating the results of the meta‐analysis are valid (Figure S8). Additionally, the pooled prevalence of RLS was not significantly altered even after the removal of any single study, further illustrating the robustness of meta‐analysis findings (Figure S9).

## 4 DISCUSSION

Currently, there is no established consensus regarding the global prevalence of RLS among HD patients. This systematic review and meta‐analysis determined the point‐in‐time prevalence of RLS in a worldwide sample of 23,248 HD patients across 21 countries. In our review, the estimated global prevalence of RLS in HD patients was 27.2% (95% CI: 24.8–29.7), which is significantly lower than the systematic review published in 2017 (Ghanei Gheshlagh et al., 2017). The source of this discrepancy could be explained by our wider subject population and larger sample size. Additionally, it is worth noting that the global prevalence of RLS among HD patients (27.2%) was remarkably higher compared to the general adult population (3%) (Broström et al., 2023). The high prevalence of RLS in HD patients may be due to the fact that reduced renal clearance and inflammatory stimuli can lead to significantly higher levels of iron‐modulating hormones in HD patients, which can cause lower serum iron levels, which in turn can impair iron utilization in the brain, leading to the incidence of RLS (Bhagawati et al., 2019; Chu et al., 2014; Sultan et al., 2022). Therefore, health care professionals should actively conduct early screening for RLS in HD patients and consider proactive interventions to reduce the incidence of adverse outcomes.

Stratified analyses based on sample size demonstrated that the prevalence of RLS in HD patients varied significantly according to each study's sample size, with studies with sample sizes below 100 reporting the highest prevalence of RLS (35.9%). In addition, meta‐regression analyses revealed that sample size was a significant moderator that may contribute to explaining the heterogeneity in the prevalence of RLS between the included studies. In general, smaller sample sizes in epidemiological studies are more likely to lead to extreme prevalence estimates (Li et al., 2020). Thus, clinical researchers should carefully consider sample size and establish an appropriate sampling strategy when conducting the study design to avoid selection bias and enhance the extrapolation of findings.

When questionnaires were utilized to diagnose RLS, the estimated overall prevalence of RLS in HD patients was 25.9%. After a detailed appraisal of each included study, we found that the majority of the questionnaires were self‐rating and without clear guidance and quality control, which may have misled participants into misinterpreting the content of the answers, resulting in a lower prevalence estimate. A previous study also suggested that when questionnaires are used as a screening tool for RLS in dialysis patients, the assessment results may be unreliable (Cirignotta et al., 2002). Among those assessed by clinical interview, a greater number of HD patients reported clinical symptoms of RLS, with a pooled prevalence of RLS of 28.7%. In the included studies, clinical interviews were mostly conducted by experienced clinicians, which gave the investigators the opportunity to provide timely and appropriate explanations when participants were in doubt and, to some extent, eliminated implementation bias.

Based on 66 studies that reported information on gender, we found that the prevalence of RLS was significantly higher in female HD patients than in male HD patients, which is consistent with earlier findings (Higuchi et al., 2015; Kim et al., 2008; Mucsi et al., 2005). However, there are also many studies stating that gender has no significant effect on RLS (Araujo et al., 2010; La Manna et al., 2011; Shi et al., 2018; Ul Abideen et al., 2018). The variation in the prevalence of RLS between male and female patients in the present study reflects the direct influence of gender on the risk of RLS, but the exact reasons for this remain unknown and may be attributed to the role of estrogens and lower levels of iron stores (Kaya et al., 2015; Rohani et al., 2015).

The stratified analysis by diagnostic criteria demonstrated that the pooled prevalence of RLS varied depending on the version of IRLSSG criteria used. The highest pooled prevalence of RLS in HD patients was reported when the diagnostic criteria were restricted to the 2003 version of IRLSSG. The 2003 version of the IRLSSG criteria is more descriptive of the symptomatic features of RLS compared to the 1995 version, thus providing greater diagnostic sensitivity, and this version has been widely used and promoted as a result (Wali & Alkhouli, 2015; Yazdi et al., 2015; Zadeh Saraji et al., 2017). Although the subsequent IRLSSG updates of 2012 and 2014 were more rigorous in terms of entries and content than the 2003 version, many researchers ignored the edition updates and persisted in adopting the 2003 version, which may therefore have contributed to the higher reported prevalence of RLS.

Subgroup analysis revealed that the prevalence of RLS varied by geographic region, with higher rates in Africa (39.0%) compared with that in Asia (25.7%), Europe (29.8%), Oceania (24.7%), and South America (30.3%). These findings contradict the results of a cross‐sectional study conducted in the United States, which reported that black dialysis patients had a lower incidence of RLS than white dialysis patients (Kutner et al., 2012), indicating that racial discrimination and geographic location may not be the only factors contributing to the variation in the prevalence of RLS and that economic status and access to primary health care should also be considered in a comprehensive manner.

In summary, our findings constitute a solid foundation for future studies of RLS secondary to HD. First, considering the potential impact of sample size on the prevalence of RLS in HD patients, sampling strategies that may encompass larger sample sizes should be employed in future studies to better ascertain the reliability and validity of prevalence estimates. Second, the strengths and differences between clinical interviews and questionnaires in collecting data related to RLS in HD patients should be further elucidated in future studies. Third, the variation in RLS prevalence between male and female HD patients should be further explored. Fourth, the RLS diagnostic criteria developed by IRLSSG are diverse and time‐spanning, and the latest version of the criteria should be prioritized in future clinical studies to ensure the accuracy of RLS diagnosis. Fifth, the overall number of relevant studies on RLS in HD patients in Oceania, the Americas, and Africa is quite small. Thus, more studies in these regions are necessary to better elucidate the impact of socioeconomic, racial, and geographic differences on the prevalence of RLS. Last but not least, the possible impact of uremic polyneuropathy on the risk of RLS in HD patients is extremely important and needs to be explored in the future by further original studies.

## 5 STRENGTHS AND LIMITATIONS

Overall, this systematic review has several notable strengths. First, we carried out an extensive search strategy without language restrictions across multiple electronic databases and applied a rigorous approach to study selection, data extraction, and appraisal. In addition, this meta‐analysis included a larger number of studies than previous meta‐analyses and used some comprehensive analysis methods to identify potential factors that contribute to RLS.

Despite the many strengths of the current study, some limitations should be acknowledged. First of all, similar to meta‐analyses of other epidemiological studies, a significantly high degree of heterogeneity was detected in this study, but except for sample size, other factors did not yield conclusive evidence to determine the possible causes of heterogeneity. Second, there was significant publication bias in the present meta‐analysis, and although the results were adjusted by trimming and padding the analyses, this may have caused the overall prevalence estimate of RLS to be lower. Third, we excluded studies that did not comply with the IRLSSG criteria from this meta‐analysis, which could have led to selection bias.

## 6 CONCLUSION

In conclusion, the current meta‐analysis demonstrated that the global prevalence of RLS in HD patients was as high as 27.2%, significantly higher than in the general population based on similar criteria. In addition, the prevalence of RLS in HD patients varied significantly depending on sample size, method of data collection, gender, diagnostic criteria, and geographical region. Additional large‐sample, multicenter studies are necessary in the future to validate and extend these prevalence findings, particularly those exploring possible associations between RLS prevalence and progression to ESRD. Last but not least, our findings may encourage clinicians to pay more attention to RLS in HD patients and support them with tailored interventions and health coaching.

## AUTHOR CONTRIBUTIONS

**Xu‐Hua Zhou**: Formal analysis; data curation; software; writing—original draft; writing—review and editing. **Yuan Liu**: Formal analysis; writing—review and editing. **Xin‐Rui Zhang**: Writing—review and editing. **Cong Wang**: Software; methodology; writing—review and editing; resources. **Shan‐Shan Liu**: Methodology; writing—review and editing. **Yan Jiang**: Conceptualization; formal analysis; writing—review and editing; software; supervision; visualization; validation; writing—original draft.

## CONFLICT OF INTEREST STATEMENT

The authors declare no conflicts of interest.

## FUNDING INFORMATION

None.

### PEER REVIEW

The peer review history for this article is available at https://publons.com/publon/10.1002/brb3.3378.

## Supporting information

Supplementary Tables Information

Supplementary section (S1)

## Data Availability

The data that support the findings of this study is available from the corresponding author upon reasonable request.
