# Supplementary material for: Global prevalence of restless legs syndrome among hemodialysis patients: A systematic review and meta‐analysis
Source: Brain Behav. 2024 Jan 11;14(1):e3378. doi: 10.1002/brb3.3378 (PMC10784193; doi:10.1002/brb3.3378)
Supplement: Supplementary file 1 — Supplementary Tables Information [file BRB3-14-e3378-s001.docx]

**Table S1.** Quality assessment of included cross-sectional studies

| First author | Item 1 | Item 2 | Item 3 | Item 4 | Item 5 | Item 6 | Item 7 | Item 8 | Item 9 | Item 10 | Item 11 | Total score |
| --- | --- | --- | --- | --- | --- | --- | --- | --- | --- | --- | --- | --- |
| Al-Jahdali et al., 2009 | 1 | 1 | 1 | 1 | 1 | 0 | 0 | 1 | 0 | 0 | 0 | 6 |
| Araujo et al., 2010 | 1 | 1 | 0 | 1 | 1 | 0 | 0 | 0 | 1 | 0 | 0 | 5 |
| Bastos et al., 2007 | 1 | 1 | 0 | 1 | 1 | 0 | 0 | 1 | 0 | 0 | 0 | 5 |
| Bathla1 et al., 2016 | 1 | 1 | 0 | 1 | 1 | 1 | 0 | 0 | 0 | 0 | 0 | 5 |
| Beladi-Mousavi et al., 2015 | 1 | 1 | 1 | 1 | 1 | 0 | 1 | 1 | 0 | 0 | 0 | 7 |
| Bhagawati et al., 2019 | 1 | 1 | 0 | 1 | 1 | 1 | 1 | 0 | 0 | 0 | 0 | 6 |
| Brzuszek et al., 2022 | 1 | 1 | 0 | 1 | 1 | 1 | 1 | 0 | 0 | 0 | 0 | 6 |
| Chavoshi et al., 2015 | 1 | 0 | 1 | 1 | 1 | 0 | 0 | 1 | 1 | 0 | 0 | 6 |
| Chen et al., 2018 | 1 | 1 | 1 | 1 | 1 | 0 | 0 | 1 | 0 | 0 | 0 | 6 |
| Chu et al., 2014 | 1 | 0 | 0 | 1 | 1 | 0 | 0 | 0 | 0 | 1 | 0 | 4 |
| Cirignotta et al., 2002 | 1 | 1 | 0 | 1 | 1 | 1 | 0 | 0 | 0 | 0 | 0 | 5 |
| Collado-Seidel et al., 1998 | 1 | 1 | 0 | 1 | 1 | 0 | 0 | 0 | 0 | 0 | 0 | 4 |
| De Menezes et al., 2018 | 1 | 1 | 1 | 1 | 1 | 0 | 0 | 1 | 0 | 0 | 0 | 6 |
| Dikici et al., 2014 | 1 | 1 | 1 | 1 | 1 | 0 | 0 | 1 | 0 | 0 | 0 | 6 |
| Du et al., 2017 | 1 | 1 | 1 | 1 | 0 | 1 | 1 | 1 | 0 | 0 | 0 | 7 |
| Giannaki et al., 2011 | 1 | 1 | 1 | 1 | 1 | 0 | 0 | 1 | 0 | 0 | 0 | 6 |
| Gigli et al., 2004 | 1 | 1 | 0 | 1 | 1 | 0 | 1 | 1 | 0 | 0 | 0 | 6 |
| Goffredo et al., 2003 | 1 | 1 | 1 | 1 | 1 | 0 | 0 | 0 | 1 | 0 | 0 | 6 |
| Guan et al., 2016 | 1 | 1 | 1 | 1 | 1 | 0 | 0 | 1 | 0 | 0 | 0 | 6 |
| Hamed et al., 2021 | 1 | 1 | 1 | 1 | 1 | 1 | 0 | 1 | 0 | 0 | 0 | 7 |
| Higuchi et al., 2015 | 1 | 1 | 1 | 1 | 1 | 0 | 0 | 1 | 0 | 0 | 0 | 6 |
| Huang et al., 2017 | 1 | 1 | 1 | 1 | 1 | 0 | 0 | 0 | 0 | 0 | 0 | 5 |
| Hui et al., 2002 | 1 | 1 | 0 | 1 | 1 | 1 | 0 | 0 | 0 | 0 | 0 | 5 |
| Hüzmeli et al., 2018 | 1 | 1 | 1 | 1 | 1 | 0 | 1 | 0 | 0 | 0 | 0 | 6 |
| Ibrahim et al., 2011 | 1 | 1 | 1 | 1 | 1 | 0 | 0 | 1 | 0 | 0 | 0 | 6 |
| Kawauchi et al., 2006 | 1 | 1 | 0 | 1 | 1 | 1 | 1 | 0 | 0 | 0 | 0 | 6 |
| Kaya et al., 2015 | 1 | 1 | 1 | 1 | 1 | 0 | 0 | 0 | 0 | 0 | 0 | 5 |
| Kim et al., 2008 | 1 | 1 | 1 | 1 | 1 | 0 | 0 | 0 | 0 | 1 | 0 | 6 |
| Kutlu et al., 2018 | 1 | 1 | 0 | 1 | 1 | 0 | 0 | 1 | 0 | 0 | 0 | 5 |
| Li et al., 2023 | 1 | 1 | 1 | 0 | 1 | 1 | 0 | 1 | 0 | 0 | 0 | 6 |
| Lin et al., 2013 | 1 | 1 | 1 | 1 | 1 | 0 | 1 | 1 | 0 | 0 | 0 | 7 |
| Lin et al., 2018 | 1 | 1 | 1 | 0 | 0 | 1 | 1 | 1 | 0 | 0 | 0 | 6 |
| Lin et al., 2019 | 1 | 1 | 1 | 1 | 1 | 1 | 0 | 1 | 1 | 0 | 0 | 8 |
| Liu et al., 2006 | 1 | 1 | 1 | 1 | 1 | 0 | 0 | 0 | 0 | 0 | 0 | 5 |
| Ma et al., 2021 | 1 | 1 | 1 | 0 | 0 | 1 | 1 | 0 | 0 | 0 | 0 | 5 |
| Meng et al., 2017 | 1 | 1 | 1 | 1 | 1 | 0 | 0 | 1 | 0 | 0 | 0 | 6 |
| Meng et al., 2021 | 1 | 1 | 1 | 0 | 0 | 1 | 0 | 0 | 0 | 0 | 0 | 4 |
| Merlino et al., 2006 | 1 | 1 | 0 | 1 | 1 | 0 | 1 | 0 | 0 | 0 | 0 | 6 |
| Merlino et al., 2012 | 1 | 1 | 1 | 1 | 1 | 0 | 0 | 0 | 0 | 0 | 0 | 5 |
| Mucsi et al., 2004 | 1 | 1 | 0 | 1 | 1 | 0 | 0 | 0 | 0 | 1 | 0 | 5 |
| Mucsi et al., 2005 | 1 | 1 | 1 | 1 | 1 | 0 | 0 | 1 | 0 | 0 | 0 | 6 |
| Naini et al., 2012 | 1 | 1 | 1 | 1 | 1 | 1 | 0 | 0 | 0 | 0 | 0 | 6 |
| Neves et al., 2017 | 1 | 1 | 0 | 1 | 1 | 0 | 1 | 1 | 0 | 0 | 0 | 6 |
| Nikić et al., 2007 | 1 | 1 | 0 | 1 | 1 | 1 | 0 | 0 | 0 | 0 | 0 | 5 |
| Örsal et al., 2017 | 1 | 0 | 1 | 1 | 1 | 0 | 0 | 0 | 0 | 0 | 0 | 4 |
| Pan et al., 2006 | 1 | 1 | 1 | 1 | 1 | 0 | 0 | 0 | 0 | 0 | 0 | 5 |
| Pavan et al., 2014 | 1 | 1 | 1 | 1 | 1 | 0 | 0 | 0 | 0 | 0 | 0 | 5 |
| Pizza et al., 2012 | 1 | 1 | 1 | 1 | 1 | 0 | 0 | 1 | 0 | 0 | 0 | 6 |
| Rafie et al., 2016 | 1 | 1 | 1 | 1 | 1 | 0 | 0 | 1 | 0 | 0 | 0 | 6 |
| Ramachandran et al., 2018 | 1 | 1 | 1 | 0 | 1 | 1 | 0 | 0 | 1 | 0 | 0 | 6 |
| Razeghi et al., 2012 | 1 | 1 | 0 | 1 | 1 | 1 | 0 | 0 | 0 | 0 | 0 | 5 |
| Rijsman et al., 2004 | 1 | 1 | 1 | 1 | 1 | 0 | 0 | 0 | 0 | 0 | 0 | 5 |
| Rohani et al., 2014 | 1 | 1 | 1 | 1 | 1 | 1 | 0 | 0 | 0 | 0 | 0 | 6 |
| Sabry et al., 2010 | 1 | 1 | 0 | 1 | 1 | 0 | 0 | 1 | 1 | 0 | 0 | 6 |
| Salman et al., 2011 | 1 | 1 | 1 | 1 | 1 | 0 | 0 | 0 | 0 | 0 | 0 | 5 |
| Samavat et al., 2017 | 1 | 1 | 1 | 1 | 1 | 0 | 0 | 1 | 0 | 0 | 0 | 6 |
| Saraji et al., 2017 | 1 | 1 | 1 | 1 | 1 | 0 | 0 | 0 | 0 | 0 | 0 | 5 |
| Shaikh et al., 2014 | 1 | 1 | 1 | 1 | 1 | 0 | 0 | 0 | 0 | 0 | 0 | 5 |
| Shao et al., 2015 | 1 | 1 | 1 | 1 | 1 | 0 | 0 | 0 | 0 | 0 | 0 | 5 |
| Shen et al., 2013 | 1 | 1 | 1 | 1 | 1 | 0 | 0 | 0 | 0 | 0 | 0 | 5 |
| Shen et al., 2018 | 1 | 1 | 1 | 1 | 1 | 0 | 0 | 1 | 0 | 0 | 0 | 6 |
| Shi et al., 2015 | 1 | 1 | 1 | 0 | 1 | 1 | 0 | 0 | 0 | 0 | 0 | 5 |
| Shi et al., 2018 | 1 | 1 | 1 | 0 | 0 | 1 | 1 | 0 | 1 | 0 | 0 | 6 |
| Siddiqui et al., 2005 | 1 | 1 | 0 | 1 | 1 | 0 | 1 | 1 | 0 | 0 | 0 | 6 |
| Sladojević et al., 2012 | 1 | 1 | 1 | 1 | 0 | 0 | 0 | 0 | 0 | 0 | 0 | 4 |
| Soumeila et al., 2015 | 1 | 1 | 1 | 1 | 1 | 0 | 0 | 0 | 0 | 0 | 0 | 5 |
| Soyoral et al., 2010 | 1 | 0 | 0 | 1 | 1 | 0 | 1 | 0 | 0 | 0 | 0 | 4 |
| Stefanidis et al., 2013 | 1 | 1 | 1 | 1 | 1 | 1 | 1 | 1 | 0 | 0 | 0 | 8 |
| Sultan et al., 2022 | 1 | 1 | 1 | 1 | 1 | 0 | 1 | 0 | 0 | 0 | 0 | 6 |
| Takaki et al., 2003 | 1 | 1 | 1 | 1 | 1 | 1 | 0 | 0 | 0 | 0 | 0 | 6 |
| Tang et al., 2014 | 1 | 1 | 1 | 1 | 1 | 1 | 0 | 0 | 1 | 1 | 0 | 7 |
| Tekdöş et al., 2015 | 1 | 1 | 1 | 1 | 1 | 0 | 0 | 0 | 0 | 0 | 0 | 5 |
| Telarović et al., 2007 | 1 | 1 | 1 | 1 | 1 | 0 | 0 | 0 | 0 | 1 | 0 | 6 |
| Tufekci et al., 2021 | 1 | 1 | 1 | 1 | 1 | 0 | 1 | 0 | 1 | 0 | 0 | 7 |
| Tuncel et al., 2011 | 1 | 1 | 0 | 1 | 1 | 0 | 0 | 0 | 0 | 0 | 0 | 4 |
| Tuo et al., 2017 | 1 | 1 | 1 | 1 | 1 | 0 | 0 | 1 | 0 | 0 | 0 | 6 |
| Turgay et al., 2018 | 1 | 1 | 1 | 1 | 1 | 0 | 0 | 0 | 0 | 0 | 0 | 5 |
| Ul Abideen et al., 2018 | 1 | 1 | 1 | 1 | 1 | 0 | 0 | 0 | 0 | 0 | 0 | 5 |
| Wali et al., 2015 | 1 | 1 | 1 | 1 | 1 | 0 | 0 | 1 | 0 | 0 | 0 | 6 |
| Wang et al., 2020 | 1 | 1 | 1 | 1 | 1 | 0 | 0 | 0 | 0 | 0 | 0 | 5 |
| Wang et al., 2023 | 1 | 1 | 1 | 1 | 1 | 1 | 1 | 0 | 0 | 1 | 0 | 8 |
| Xiao et al., 2013 | 1 | 1 | 0 | 1 | 1 | 1 | 0 | 0 | 0 | 0 | 0 | 5 |
| Xiao et al., 2017 | 1 | 1 | 1 | 1 | 1 | 0 | 0 | 1 | 0 | 0 | 0 | 6 |
| Xu et al., 2015 | 1 | 1 | 0 | 1 | 1 | 1 | 1 | 0 | 0 | 0 | 0 | 6 |
| Xu et al., 2023 | 1 | 1 | 1 | 1 | 0 | 0 | 0 | 1 | 0 | 1 | 0 | 6 |
| Yaseen et al., 2022 | 1 | 1 | 1 | 1 | 1 | 0 | 0 | 0 | 0 | 0 | 0 | 5 |
| Yazdi et al., 2015 | 1 | 0 | 1 | 1 | 1 | 0 | 0 | 0 | 0 | 0 | 0 | 4 |
| Zaware et al., 2016 | 1 | 1 | 1 | 0 | 0 | 1 | 0 | 0 | 0 | 0 | 0 | 5 |
| Zeng et al., 2022 | 1 | 1 | 1 | 0 | 0 | 1 | 0 | 0 | 0 | 1 | 0 | 6 |
| Zhang et al., 2007 | 1 | 1 | 1 | 1 | 1 | 0 | 0 | 0 | 0 | 0 | 0 | 5 |
| Zhang et al., 2016 | 1 | 1 | 1 | 1 | 1 | 0 | 0 | 1 | 0 | 0 | 0 | 6 |
| Zhang et al., 2020 | 1 | 1 | 0 | 1 | 1 | 1 | 1 | 1 | 0 | 0 | 0 | 7 |
| Zhang et al., 2022 | 1 | 1 | 1 | 1 | 0 | 1 | 0 | 0 | 0 | 0 | 0 | 6 |
| Zhang et al., 2022 | 1 | 1 | 0 | 1 | 1 | 1 | 0 | 0 | 0 | 0 | 0 | 5 |
| Zhong et al., 2012 | 1 | 1 | 0 | 1 | 1 | 0 | 0 | 1 | 0 | 0 | 0 | 5 |

**Table S2.** Quality assessment of included cohort studies

| First author | Item 1 | Item 2 | Item 3 | Item 4 | Item 5 | Item 6 | Item 7 | Item 8 | Total score |
| --- | --- | --- | --- | --- | --- | --- | --- | --- | --- |
| La Manna et al., 2011 | 1 | 1 | 1 | 0 | 1 | 1 | 1 | 1 | 7 |
| Yang et al., 2019 | 1 | 1 | 1 | 0 | 2 | 1 | 1 | 1 | 8 |


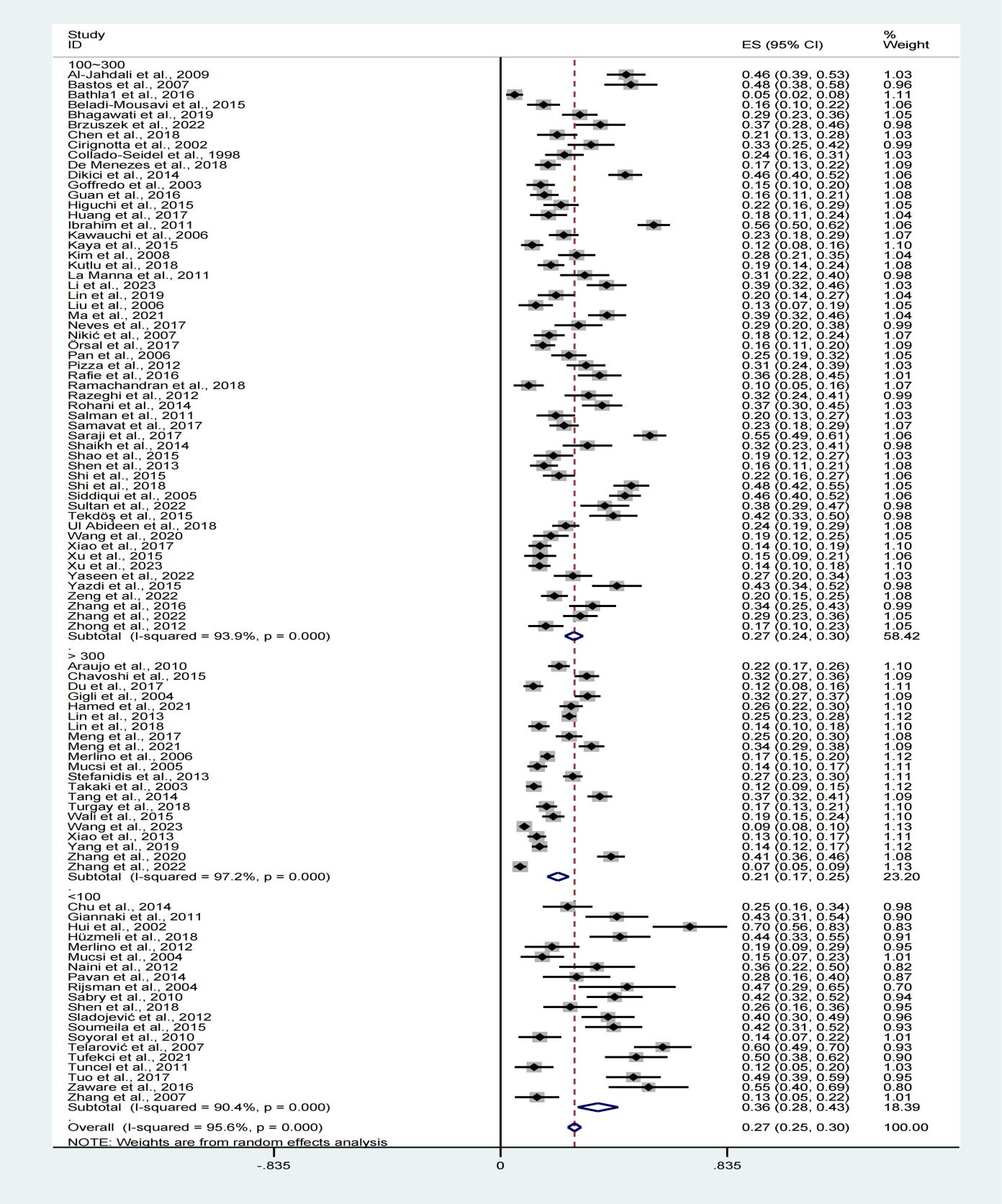


**Figure S1.** Forest plot of pooled prevalence of restless legs syndrome among hemodialysis patients based on sample size


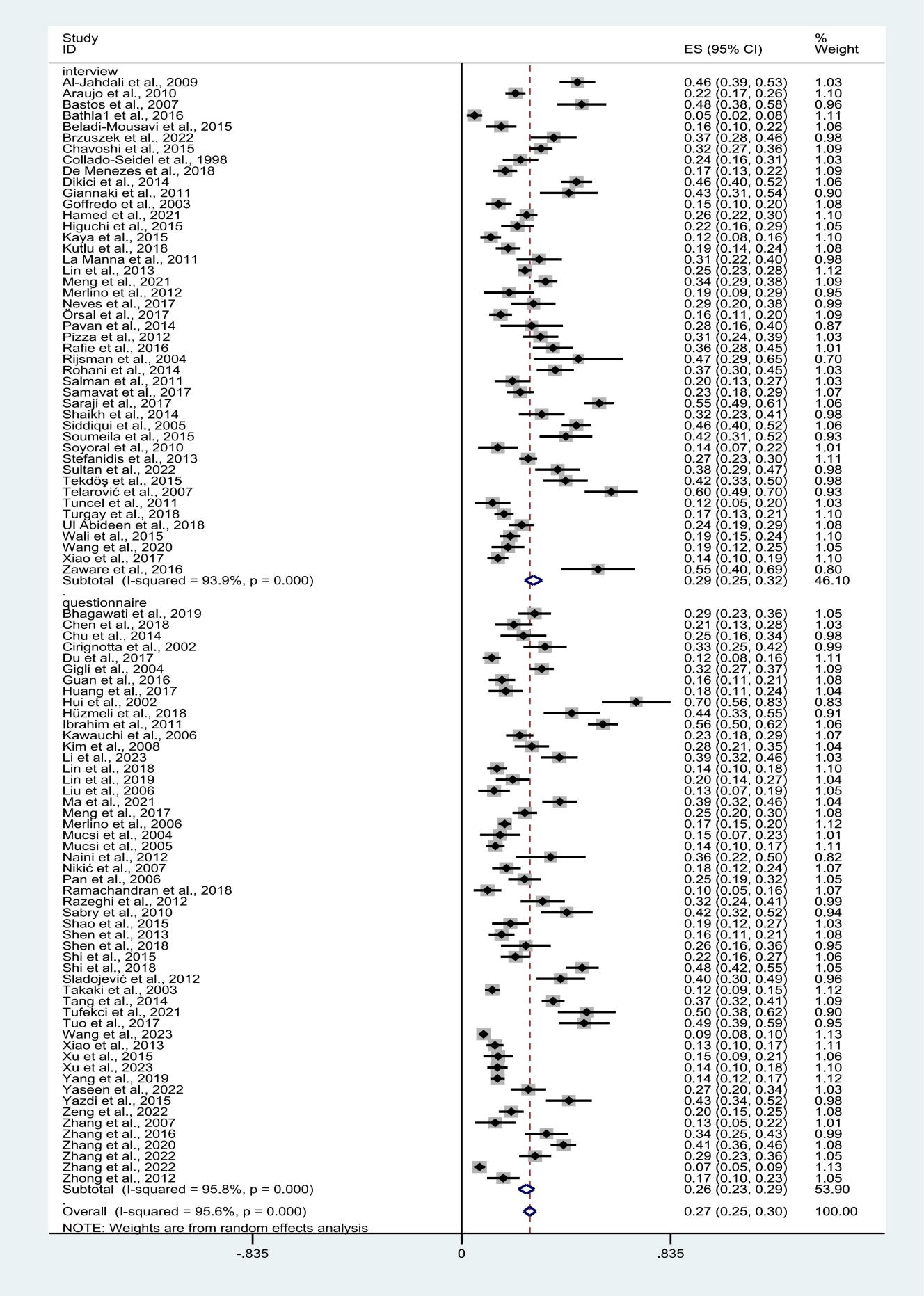
**Figure S2.** Forest plot of pooled prevalence of restless legs syndrome among hemodialysis patients based on data collection method


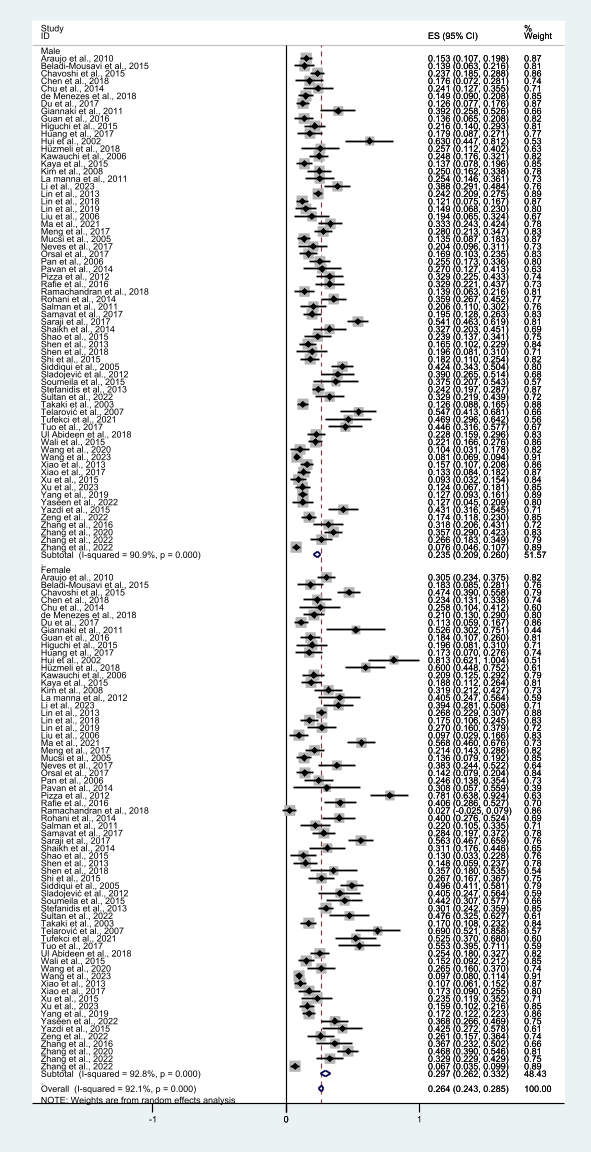


**Figure S3.** Forest plot of pooled prevalence of restless legs syndrome among hemodialysis patients based on gender


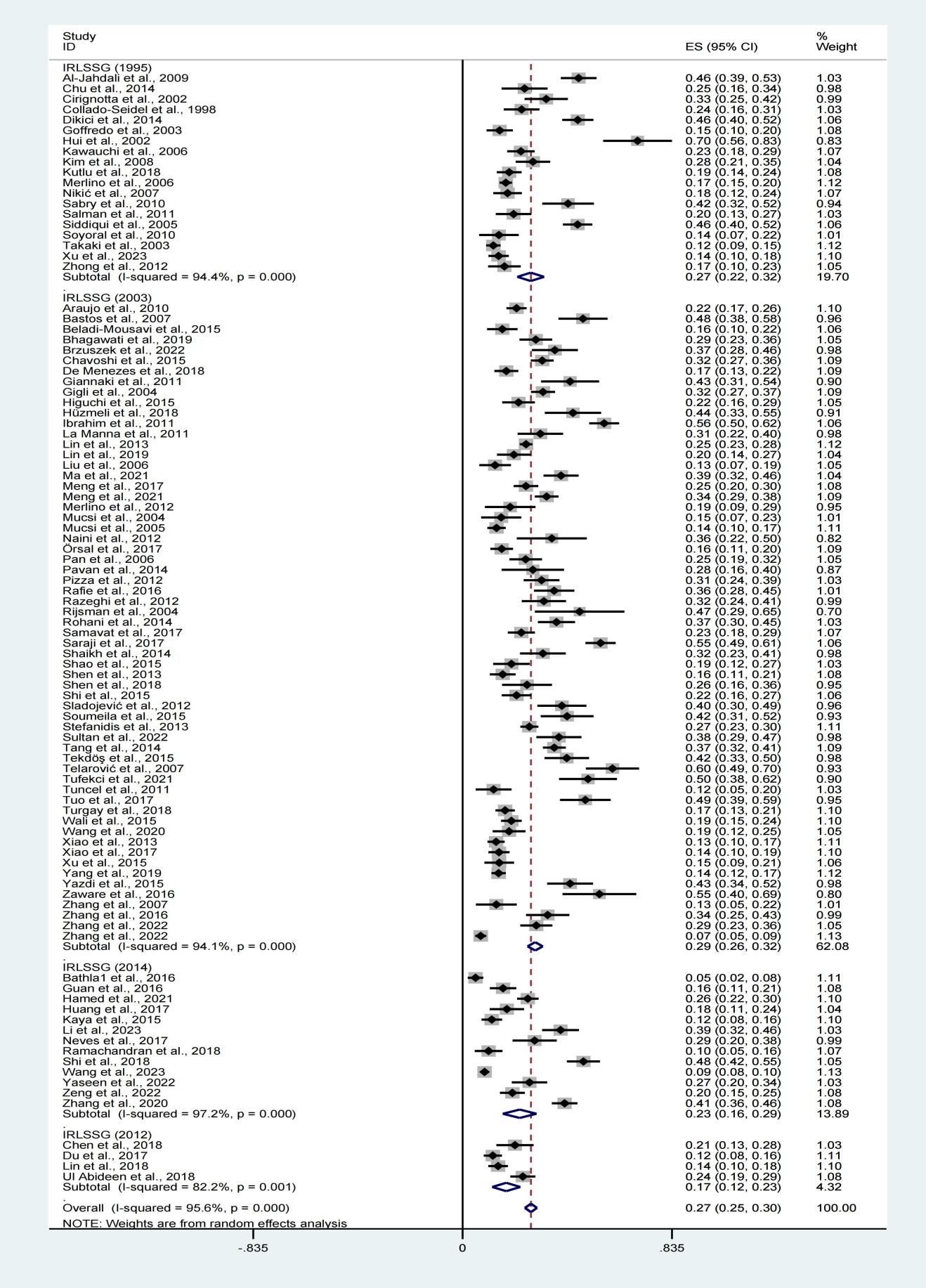


**Figure S4.** Forest plot of pooled prevalence of restless legs syndrome among hemodialysis patients based on diagnostic criteria


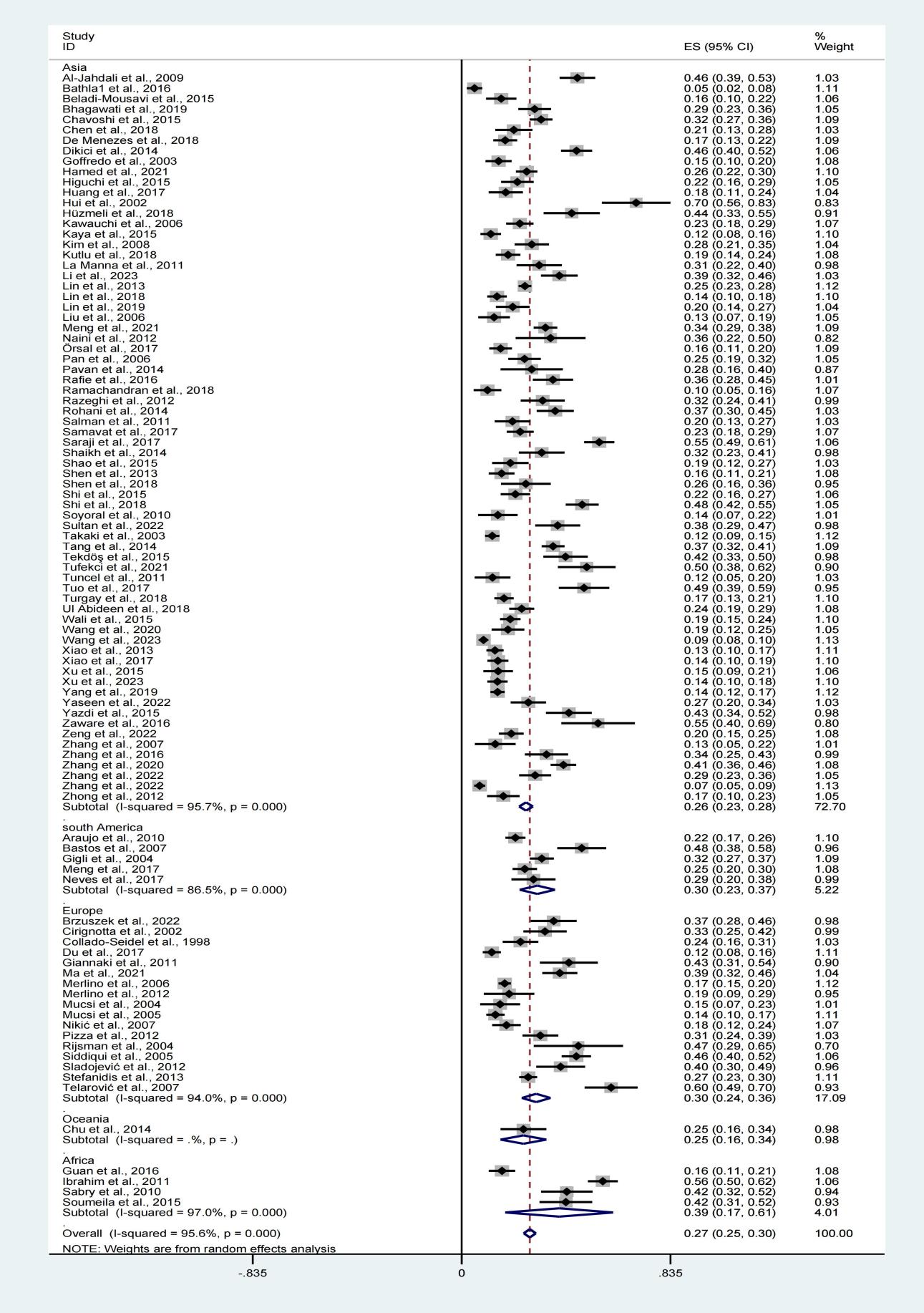


**Figure S5.** Forest plot of pooled prevalence of restless legs syndrome among hemodialysis patients based on geographical region


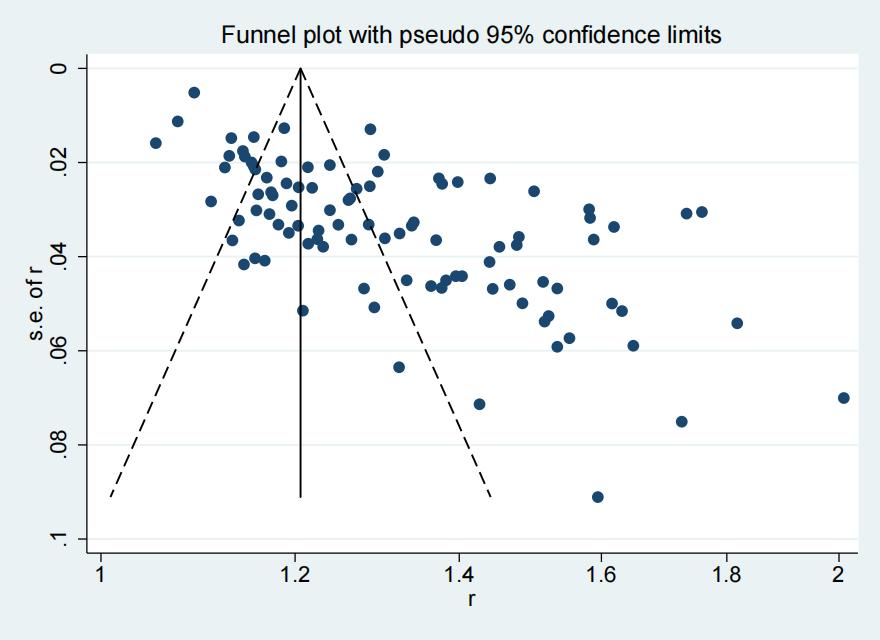


**Figure S6.** Funnel plot of pooled prevalence of restless legs syndrome among hemodialysis patients


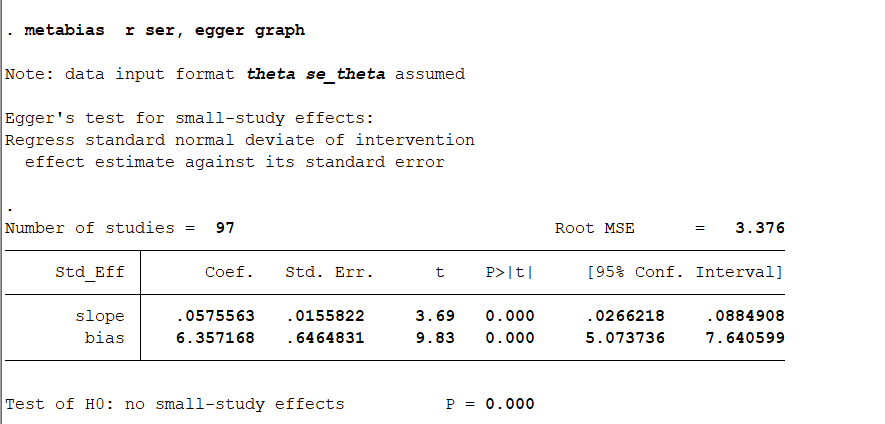


**Figure S7.** Egger's test results graph


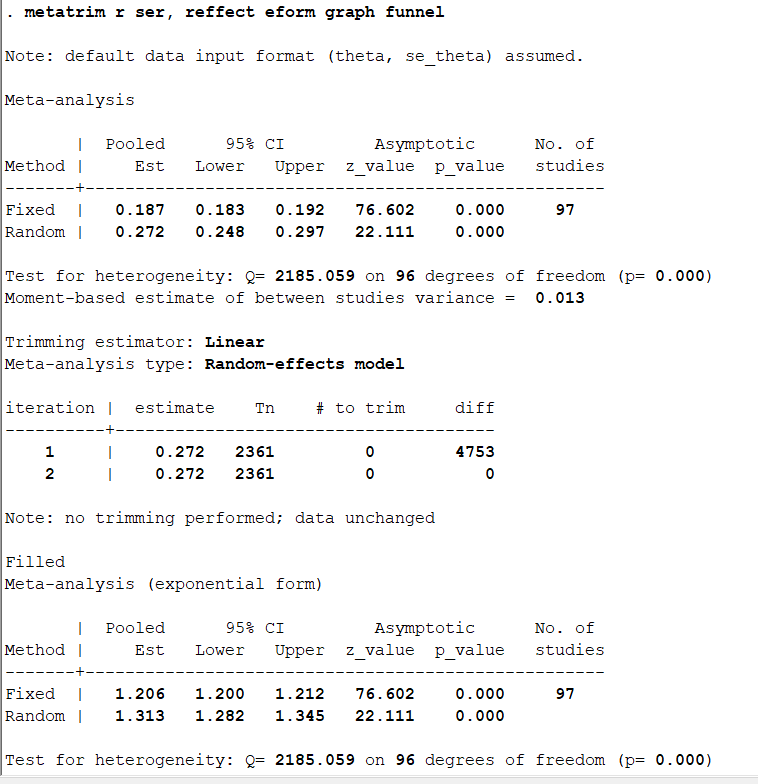


**Figure S8.** Trim-fill analysis results

**Table S3** Results of univariate and multivariate meta-regression analysis of restless legs syndrome

|  | **B** | **95%CI** | **t** | **p** | **Adjusted R^2^** |
| --- | --- | --- | --- | --- | --- |
| **Univariate** |  |  |  |  |  |
| Sample size | -0.07 | -0.11, -0.03 | -3.55 | 0.001 | 10.83% |
| Data collection method | -0.03 | -0.08, 0.03 | -1.02 | 0.312 | 0.08% |
| Gender | 0.0006 | -0.051, 0.009 | 1.03 | 0.307 | 0.13% |
| Diagnostic criteria | -0.02 | 0.05, 0.25 | -1.4 | 0.166 | 0.95% |
| Geographic region | -0.005 | -0.04, 0.03 | -0.29 | 0.770 | -1.07% |
| **Multivariate** |  |  |  |  |  |
| Sample size | -0.07 | -0.11, 0.02 | -3.17 | 0.002 | 8.94% |
| Data collection method | -0.03 | -0.08, 0.03 | -0.95 | 0.812 |  |
| Gender | -0.0003 | -.0008, 0.0014 | -1.98 | 0.059 |  |
| Diagnostic criteria | -0.01 | -0.04, 0.01 | -0.84 | 0.403 |  |
| Geographic region | -0.01 | -0.04, 0.02 | -0.64 | 0.014 |  |


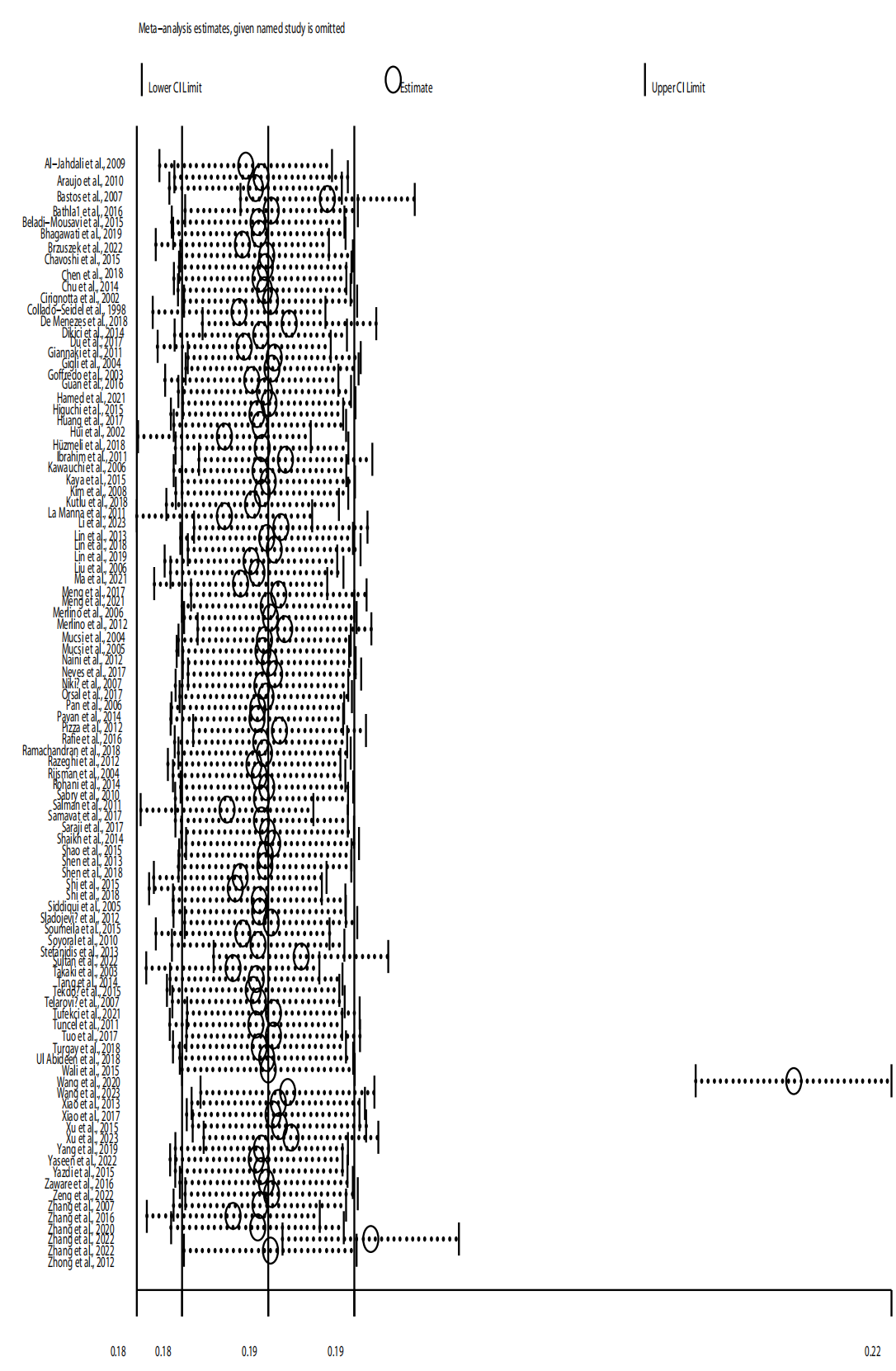
**Figure S9.** Sensitivity analysis of pooled prevalence of restless legs syndrome among hemodialysis patients
