# Supplementary material for: Global prevalence of restless legs syndrome among hemodialysis patients: A systematic review and meta‐analysis
Source: Brain Behav. 2024 Jan 11;14(1):e3378. doi: 10.1002/brb3.3378 (PMC10784193; doi:10.1002/brb3.3378)
Supplement: Supplementary file 2 — Supplementary section (S1) [file BRB3-14-e3378-s002.docx]

**supplementary section (S1)**

1. **Cochrane Library Search Strategy**

Search Date: August 10, 2023

Final Search:#7

| **Search** | **Query** | **No. of Results** |
| --- | --- | --- |
| **#1** | MeSH descriptor: [Dialysis] explode all trees | 750 |
| **#2** | (“dialysis”):ti,ab,kw OR (“hemodialysis”):ti,ab,kw | 21982 |
| **#3** | #1 OR #2 | 22204 |
| **#4** | MeSH descriptor: [Restless Legs Syndrome] explode all trees | 510 |
| **#5** | (“Willis Ekbom Disease” OR “Wittmaack-Ekbom Syndrome”):ti,ab,kw | 21 |
| **#6** | #4 OR #5 | 516 |
| **#7** | #3 AND #6 | 51 |

1. **CINAHL Search Strategy (CINAHL Plus with Full Text)**

Search Date: August 10, 2023

Final Search: S3

| **Search** | **Query** | **No. of Results** |
| --- | --- | --- |
| **S1** | SU dialysis OR hemodialysis | 31247 |
| **S2** | SU Restless Legs Syndrome OR Willis Ekbom Disease OR Wittmaack-Ekbom Syndrome | 236 |
| **S3** | S1 AND S2 | 7 |

1. **PubMed Search Strategy**

Search Date: August 10, 2023

Final Search: #7

| **Search** | **Query** | **No. of Results** |
| --- | --- | --- |
| **#1** | "dialysis"[Mesh Terms] | 24115 |
| **#2** | "dialysis"[Title/Abstract] OR "hemodialysis"[Title/Abstract] | 166888 |
| **#3** | #1 OR #2 | 184927 |
| **#4** | "Restless Legs Syndrome"[MeSH Terms] | 4302 |
| **#5** | "restless legs syndrome"[Title/Abstract] OR "Willis Ekbom Disease"[Title/Abstract] OR "Wittmaack-Ekbom Syndrome"[Title/Abstract] | 4688 |
| **#6** | #4 OR #5 | 5634 |
| **#7** | #3 AND #6 | 287 |

1. **Embase Search Strategy**

Search Date: August 10, 2023

Final Search: #8

| **Search** | **Query** | **No. of Results** |
| --- | --- | --- |
| **#1** | 'dialysis'/exp | 34597 |
| **#2** | 'hemodialysis'/exp | 139833 |
| **#3** | 'dialysis':ti,ab,kw OR 'hemodialysis':ti,ab,kw | 250493 |
| **#4** | #1 OR #2 OR #3 | 358398 |
| **#5** | 'restless legs syndrome'/exp | 11963 |
| **#6** | 'restless legs syndrome':ti,ab,kw OR 'Willis Ekbom Disease':ti,ab,kw OR 'Wittmaack-Ekbom Syndrome':ti,ab,kw | 7530 |
| **#7** | #5 OR #6 | 12436 |
| **#8** | #4 AND #7 | 576 |

1. **Web of Science Search Strategy**

Search Date: August 10, 2023

Final Search: #3

| **Search** | **Query** | **No. of Results** |
| --- | --- | --- |
| **#1** | TS=("dialysis" OR "hemodialysis") | 401032 |
| **#2** | TS=("restless legs syndrome" OR "Willis Ekbom Disease" OR "Wittmaack-Ekbom Syndrome") | 12828 |
| **#3** | #1 AND #2 | 625 |

1. **Scopus Search Strategy**

Search Date: August 10, 2023

Final Search: #3

| **Search** | **Query** | **No. of Results** |
| --- | --- | --- |
| **#1** | TITLE-ABS-KEY ( "dialysis"  OR  "hemodialysis" ) | 288672 |
| **#2** | TITLE-ABS-KEY ( "restless legs syndrome"  OR  "Willis Ekbom Disease"  OR  "Wittmaack-Ekbom Syndrome") | 10393 |
| **#3** | #1 AND #2 | 570 |

**G. CNKI Search Strategy**

Search Date: August 10, 2023

| (主题="血液透析" + "维持性血液透析" + "常规血液透析"+ "血液净化治疗") [AND (主题="不宁腿综合征"+"不安腿综合征")](https://kns.cnki.net/KNS8/AdvSearch?id=615&dbcode=SCDB&searchtype=gradeSearch&ishistory=1" \t "_blank" \o "(主题="乳腺癌" + "乳房癌" + "乳房肿瘤" + "乳腺肿瘤"  + "乳腺恶性肿瘤") AND (主题="衰弱" + "虚弱" + "衰弱症" + "衰弱综合征"))/258 |
| --- |

**H. VIP Search Strategy**

Search Date: August 10, 2023

| [(题名或关键词="血液透析" OR "维持性血液透析" OR "常规血液透析" OR "血液透析治疗") AND (题名或关键词="不宁腿综合征" OR "不安腿综合征")](http://lib.cqvip.com/Qikan/search/index?LngMySearHistoryIdGuid=35f85ed6-00e0-4eee-b999-17896f9298ec&from=Qikan_Article_History)/215 |
| --- |

**I. WAN FANG Search Strategy**

Search Date: August 10, 2023

| 题名或关键词:(血液透析OR 维持性血液透析 OR 常规血液透析 OR 血液透析治疗) and 题名或关键词:(不宁腿综合征 OR 不安腿综合征)/230 |
| --- |

**J. CBM Search Strategy**

Search Date: August 10, 2023

| [("血液透析"[常用字段:智能] OR "维持性血液透析"[常用字段:智能] OR "常规血液透析"[常用字段:智能] OR "血液透析治疗"[常用字段:智能]) AND ("不宁腿综合征"[常用字段:智能] OR "不安腿综合征"[常用字段:智能])](javascript:toDoRelimitSearch();)/304 |
| --- |
